# Incorporating geography into a new generalized theoretical and statistical framework addressing the modifiable areal unit problem

**DOI:** 10.1186/s12942-019-0170-3

**Published:** 2019-03-27

**Authors:** M. Tuson, M. Yap, M. R. Kok, K. Murray, B. Turlach, D. Whyatt

**Affiliations:** 1 School of Mathematics, Physics, and Computing, University of Western Australia, Perth, Australia; 2 Medical School, University of Western Australia, Perth, Australia; 3 School of Population and Global Health, University of Western Australia, Perth, Australia

**Keywords:** Modifiable areal unit problem, Automated zonation construction, AZTool, Areal units, Estimation of associations

## Abstract

**Background:**

All analyses of spatially aggregated data are vulnerable to the modifiable areal unit problem (MAUP), which describes the sensitivity of analytical results to the arbitrary choice of spatial aggregation unit at which data are measured. The MAUP is a serious problem endemic to analyses of spatially aggregated data in all scientific disciplines. However, the impact of the MAUP is rarely considered, perhaps partly because it is still widely considered to be unsolvable.

**Results:**

It was originally understood that a solution to the MAUP should constitute a comprehensive statistical framework describing the regularities in estimates of association observed at different combinations of spatial scale and zonation. Additionally, it has been debated how such a solution should incorporate the geographical characteristics of areal units (e.g. shape, size, and configuration), and in particular whether this can be achieved in a purely mathematical framework (i.e. independent of areal units). We argue that the consideration of areal units must form part of a solution to the MAUP, since the MAUP only manifests in their presence. Thus, we present a theoretical and statistical framework that incorporates the characteristics of areal units by combining estimates obtained from different scales and zonations. We show that associations estimated at scales larger than a minimal geographical unit of analysis are systematically biased from a true minimal-level effect, with different zonations generating uniquely biased estimates. Therefore, it is fundamentally erroneous to infer conclusions based on data that are spatially aggregated beyond the minimal level. Instead, researchers should measure and display information, estimate effects, and infer conclusions at the smallest possible meaningful geographical scale. The framework we develop facilitates this.

**Conclusions:**

The proposed framework represents a new minimum standard in the estimation of associations using spatially aggregated data, and a reference point against which previous findings and misconceptions related to the MAUP can be understood.

**Electronic supplementary material:**

The online version of this article (10.1186/s12942-019-0170-3) contains supplementary material, which is available to authorized users.

## Background

The modifiable areal unit problem (MAUP) has long been considered one of the most important unresolved problems in spatial analysis [1]. First identified in 1934 [2], with the term coined by geographers in 1979 [3], the MAUP describes the sensitivity of analytical results to the arbitrary choice of spatial aggregation unit at which data is measured (e.g. census tracts or raster cells) [1, 4]. Summary values (e.g. counts) can be dramatically affected by both the position of boundaries between areal units (the zonation aspect) and the scale of the aggregation unit (the scale aspect), and estimates of association between variables (e.g. correlation coefficients) based on areal data are similarly affected [1, 4].

The consequence of the MAUP is that estimates obtained, and conclusions inferred, from analyses of data aggregated at a single zonation, are dependent on the chosen aggregation unit [1, 5, 6]. This is a serious problem undermining analyses of spatially aggregated data in all scientific disciplines, including ecological studies [7], epidemiology and health research [8, 9], demography [10], the design of electoral boundaries [11], economics [12], transport and traffic modelling [13], criminology [14], and physical geography [15], among many others.

Unfortunately, the impact of the MAUP is rarely considered; a recent review found the MAUP to be acknowledged in only about 1% of publications using spatially aggregated data [16]. It is possible that in many cases this may be due to ignorance of the MAUP. However, it has also been suggested that the MAUP may be ignored because to acknowledge it is to question the validity of any conclusions drawn from analyses of spatially aggregated data (including one’s own) [16]. That the MAUP is still widely considered to be unsolvable may have exacerbated this situation [1, 16]. Nevertheless, to take such an approach, despite it being noted 34 years ago that: “the widespread and serious impact of the MAUP on spatial study has been convincingly demonstrated so it is no longer possible to simply ignore it” [1], is regrettable. In the absence of a generalized solution, sensitivity analyses investigating estimates at multiple scales and zonations are the minimum standard to examine the MAUP’s impact [4, 5], but these are also rarely undertaken.

In estimating associations between variables, it was originally understood that a solution to the MAUP should constitute a comprehensive statistical framework describing the regularities in estimates observed at different combinations of spatial scale and zonation [1, 3, 17]. Additionally, it has been argued that critical to such a solution would be the incorporation of the geographical characteristics of areal units (e.g. shape, size, and configuration) [18, 19]. However, to our knowledge, no generalized method achieving this has been developed. To address this need, we examine the impact of the MAUP in univariate and multivariate contexts. Emphasis is placed on the latter, wherein we develop a novel statistical framework to quantify the uncertainty induced by the MAUP in the estimation of associations. This framework exploits the MAUP by combining estimates obtained at different scales and zonations, thereby incorporating the geographical characteristics of the data.

## Results

### Addressing the MAUP in a univariate context

**Fig. 1:**
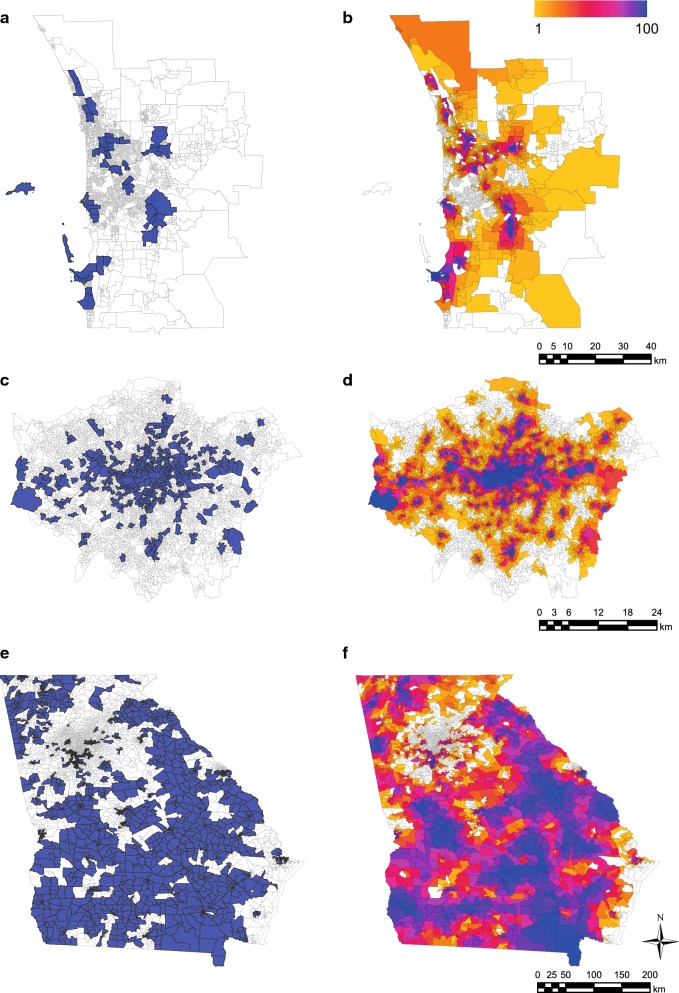
Overlaying multiple zonations improves choropleth map accuracy. **a** SA2s with MH ED presentation rates statistically higher than average in Perth. **b** Number of times each SA1 is comprised in a high-rate area across 100 zonations. **c** MSOAs with crime rates statistically higher than average in London. **d** Number of times each LSOA is comprised in a high-rate area across 100 zonations. **e** Areas with mean income statistically lower than average in Georgia. **f** Number of times each CBG is comprised in a low-income area across 100 zonations. The areas in **e** correspond to the first zonation out of the 100 underlying (**f**). In **b**, **d**, **f**, areas are colored from yellow (one) to blue (100), with white areas representing zero

Figure 1 illustrates the MAUP in the context of choropleth mapping of a single variable (i.e. in a univariate context), using real-world data. Figure 1a shows areas with rates of presentations to hospital emergency departments with mental health-related diagnoses (MH ED presentations) among residents that are statistically higher than average (‘high-rate’ areas), in Perth, Western Australia. The displayed areas are Statistical Area Level 2 (SA2) administrative regions, which have a mean population size of approximately 10,000 and comprise contiguous minimal areal units (Statistical Area Level 1 (SA1) regions) [20]. SA1s have a mean population size of approximately 430 residents. In contrast, Fig. 1b overlays high-rate areas from 100 different zonations, each constructed by aggregating contiguous SA1s to a target population size of 10,000 residents. These zonations were constructed using the freeware AZTool (see “Methods” for a description of this software and its implementation). Each SA1 is colored from yellow to blue according to the number of zonations in which it was comprised in a high-rate area, with white areas representing zero.

Similar maps are shown for two other geographies: London (UK) and Georgia (USA). Figure 1c shows Middle Layer Super Output Areas (MSOAs) with higher than average crime rates [21] (‘high-rate’ areas) in London, and Fig. 1d overlays high-rate areas from 100 different zonations constructed by contiguously aggregating a minimal areal unit [lower layer super output areas (LSOAs); mean population size 1700] to a target population size of 8000. Figure 1e shows areas with lower than average mean income (‘low-income’ areas) in Georgia, and Fig. 1f overlays low-income areas from 100 different zonations constructed by contiguously aggregating a minimal areal unit [census block groups (CBGs); mean population size 1750; 22] to a target population size of 8000. The areas in Fig. 1e correspond to the first zonation out of the 100 underlying Fig. 1f.

In Fig. 1, minimal units in high-rate or low-income areas in a particular zonation have an average probability of being similarly identified in another zonation of 65% (95% quantile range 61–69), 65% (63–66), and 74% (73–75) for Perth, London, and Georgia, respectively. The unreliability of single-zonation maps has been comprehensively demonstrated in the literature (e.g. see [1]), though it has not previously been quantified in this manner. Unfortunately, however, such maps are still frequently constructed with impunity. Here we note that the impact of the MAUP in choropleth mapping could be managed by overlaying numerous zonations, as we have done. This approach is not developed further here, but is suggested as the basis for an alternative to existing smoothing and geostatistical areal interpolation methods to overcome the MAUP in a univariate context (e.g. see [23, 24]). In the following section we use the same zonations underlying Fig. 1, combined with equivalent zonations constructed at other scales, to develop a framework to quantify the uncertainty induced by the MAUP in a multivariate context.

### Addressing the MAUP in a multivariate context

#### Illustration using simulated data

**Fig. 2:**
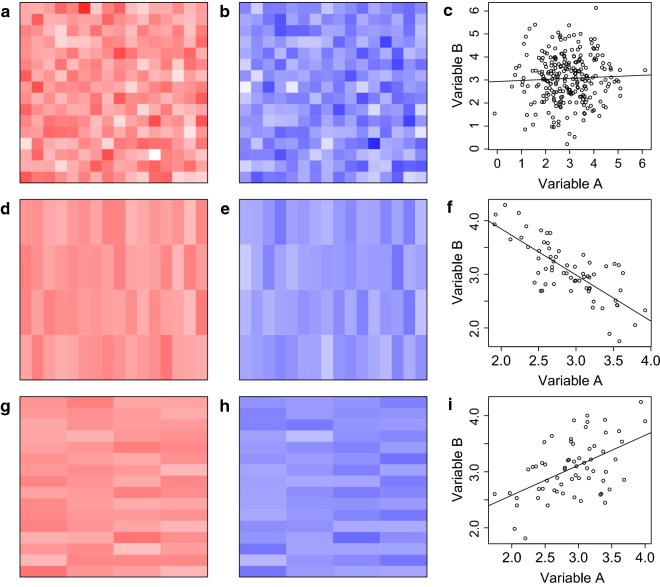
Illustrating the MAUP using simulated data. **a**, **b** Data for variables $A$ (red) and $B$ (blue) at a minimal unit of analysis. **d**, **e** Shown at one higher-scale zonation. **g**, **h** Shown at a second higher-scale zonation. **c**, **f**, **i** The linear relationship between $A$ and $B$ at the minimal level and at the first and second higher-scale zonations, respectively, with fitted regression lines

Figure 2 (modified from [25]) illustrates the zonation and scale aspects of the MAUP in a multivariate context, using simulated data. The distributions of an independent variable $A$ and a dependent variable $B$ are shown at a minimal areal unit of analysis (Fig. 2a, b) and at two different higher-scale zonations (Fig. 2d, e, g, h). The estimated association between $A$ and $B$ is not statistically significant at the minimal level (*p* = 0.486; Fig. 2c), significant and negative at the first higher-scale zonation (β = − 0.86, *p* < 0.001; Fig. 2f), and significant and positive at the second higher-scale zonation (β = 0.535, *p* < 0.001; Fig. 2i). Clearly, the estimated association at different higher-scale zonations may be substantially affected by the choice of aggregation unit, in this case changing from negative to positive.

#### Illustration using real-world data

**Fig. 3:**
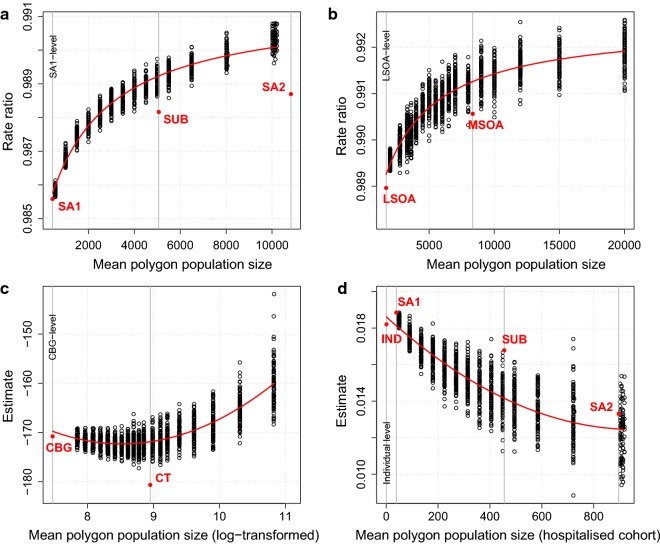
MAUP in different geographies. **a** Estimated RRs of MH ED presentations associated with a unit increase in IRSAD percentile, at combinations of scale and zonation in Perth. **b** Estimated RRs of crime associated with a unit increase in socioeconomic percentile in London. **c** Estimated mean increase in mean Charlson comorbidity index associated with a 1 year increase in mean age in Perth. **d** Estimated mean decrease in income associated with a 1% increase in percentage black population in Georgia. Fitted lines; estimates from arbitrary administrative zonations [suburb (SUB) and SA2 in **a**; MSOA in **b**; Census Tracts (CTs) in **c**; SUB and SA2 in **d**]; and minimal level estimates [SA1 in **a**; LSOA in **b**; CBG in **c**; and individual in **d**] are shown

Using real-world data, Fig. 3a shows rate ratios (RRs) of MH ED presentations associated with a unit increase in a socioeconomic percentile [26] in Perth. Open circles represent estimates derived from negative binomial (NB) models fitted at different zonations at different scales. The estimated effect sizes vary substantially, with the largest being 56% greater than the smallest. However, the systematic nature of the variation suggests a regression line could be fitted. Such a line is shown, along with estimates at arbitrary administrative zonations, including the minimal SA1-level estimate. Interestingly, the latter estimate lies close to an extrapolated point from the fitted line. However, as we show later, this is not always the case.

Figure 3b–d illustrate similar systematic variation in association estimates in three other examples. Figure 3b shows RRs derived from NB models regressing crime against a socioeconomic deprivation percentile [27] in London; Fig. 3c shows estimates from standard linear models regressing income against the percentage of black individuals [22] in Georgia; and Fig. 3d shows estimates from standard linear models regressing the mean Charlson co-morbidity index [28] against mean age among individuals in Perth who had been previously hospitalized. The effect sizes vary from smallest to largest by 45%, 20% and 113%, respectively. In each plot, estimates at arbitrary administrative zonations are shown, and fitted curves are extrapolated to points close to estimates derived at the minimal level (LSOAs, CBGs and individuals, respectively). The latter example illustrates the application of methods introduced below to cases where the minimal unit of analysis is non-areal (in this case, individual).

#### A new approach

Considering the extensive literature examining the MAUP, it is remarkable that regression lines have not previously been fitted to sets of association estimates such as those in Fig. 3, despite systematic variation in such estimates being previously observed [5, 8, 9]. Building on this observation, we introduce a method to identify the range of minimal-level values (‘true effects’) that could have generated an observed set of estimates (and a corresponding fitted line). These ‘simulation intervals’ (SIs) are derived using repeated data simulation at the minimal level. Analogously to confidence intervals (CIs), a particular SI will, by construction, comprise the true, minimal-level effect underlying a set of estimates with asymptotic coverage equal to a desired level (e.g. 95%). Importantly, note that the construction of SIs in this manner is necessary because the extrapolated intercept of a fitted line at the minimal level (denoted $EI_{\beta }$) is not directly an estimate of an effect $\beta$ at that scale, but merely an interim value used in constructing a SI for $\beta$ (see “Methods” and Additional file 1: Figs. S1–S3). Furthermore, this method relies on the ability to simulate data at a minimal level after assuming an underlying model structure at that level. Such assumptions underpin all statistical analyses, and may dictate univariate or multivariate models, interactions between independent variables, or the presence of spatial autocorrelation, for example.

**Fig. 4:**
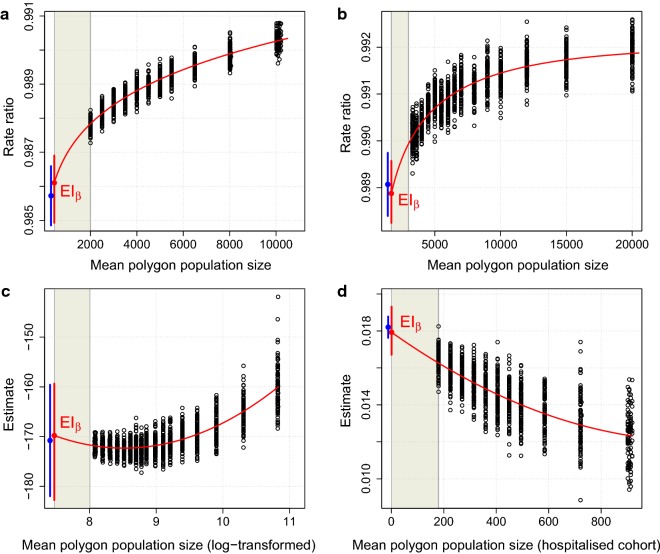
Simulation intervals for real-world data. **a**–**d** Data in Fig. 3 re-presented under stopping point population sizes of 2000 (**a**); 3000 (**b**); 3000 (**c**); and 2000 (**d**), respectively. Fitted lines; extrapolated intercepts ($EI_{\beta }$ values); and 95% SIs are shown (red), along with minimal-level estimates (SA1 in **a**; LSOA in **b**; CBG in **c**; and individual in **d**) and their 95% CIs (blue). Shaded regions indicate scales where estimates were intentionally omitted

Here we illustrate the construction of 95% SIs for real-world data. Figure 4 (see also Additional file 1: Figs. S4–S7) re-presents data in Fig. 3 after restricting each example according to hypothetical ‘stopping point’ population sizes of 2000 (Fig. 4a, d) or 3000 individuals (Fig. 4b, c). Such restrictions frequently operate in practice, for example when events are rare, and limit the ability to fit models at smaller scales or at the minimal level. This point may be more pertinent when complex models are fitted, for example models with many covariates and complex correlation structures, since such models often fail to converge when fitted to sparse datasets with small counts. Figure 4 shows regression lines, $EI_{\beta }$ values, and 95% SIs at the minimal level, for each example. Also shown are minimal-level estimates and their 95% CIs, although in practice these may not be obtainable due to the (stopping-point) restrictions noted. The two intervals are presented together for comparative purposes; the fact that the 95% SIs largely overlap the 95% CIs, despite being constructed without access to model estimates at scales smaller than their corresponding stopping points, demonstrates their utility.

## Discussion

We have shown that differences in association estimates derived from data aggregated to different zonations and at different scales (i.e. the MAUP) can be recreated using a range of values operating at a minimal areal unit of analysis. This may seem self-evident, once observed, but it has not been previously demonstrated. Furthermore, this represents a solution to the MAUP in the estimation of associations, according to the original understanding of what such a solution should comprise [1, 3, 17]. Therefore, all aggregate-level estimates are systematically biased from a true minimal-level effect, and aggregate-level CIs will not have asymptotic coverage of the true effect, except possibly by chance. Consequently, it is fundamentally erroneous to infer an association between two or more variables based on data that are spatially aggregated beyond the minimal level. These findings suggest a new minimum standard for the estimation of associations using spatially aggregated data: this should be done either directly at the minimal level or using the simulation framework we have developed.

The concept of a minimal geographic unit has been described elsewhere, for example as ‘basic areal units’ in [29]. The minimal unit may be determined by technology (e.g. the smallest raster cell size at which data can be measured), by process (e.g. when only pre-defined minimal administrative boundaries are available), or by the fact that to measure at a finer scale would not be meaningful [30]. An example of the latter case might be examining the relationship between landscape heterogeneity and amphibian biodiversity using watersheds as a minimal unit [31]. In this manner the minimal areal unit may be deemed unmodifiable. However, when higher resolution data are meaningful, but not available, what is referred to as the true effect at the minimal level is provisionary until such data becomes available. It is worth reiterating the case where only data aggregated to fixed administrative units are available, since this frequently occurs in practice and analyses of such data are often relied upon to guide the allocation of scarce resources (e.g. in health care). As noted above, in such cases the minimal unit is defined by process, and the analyst is usually powerless to influence this. Nevertheless, results from such analyses should be accompanied by a statement regarding their dependence on the units used.

In some cases, the minimal areal unit may remain modifiable. This situation is perhaps best understood in physical geography, where numerous minimal-level zonations may be obtained by shifting a raster grid by an amount less than the width of a minimal cell. An example of this was observed in [32], where the authors proposed averaging results based on multiple orientations of minimal-level square cell grids. However, in such cases, no single true effect is shared between differing minimal-level zonations, since each zonation will create a unique minimal-level joint distribution. Consequently, no single minimal-level effect can be interpreted as being absolutely true, and minimal-level SIs or CIs will only have asymptotic coverage of the true effect specific to the chosen minimal-level zonation. Therefore, it is important to maintain cognizance of both the variability in such estimates, and the uncertainty associated with each estimate. Thus, there is no justification for relying on either: one out of a group of minimal-level estimates, or a summary measure of the group. Consequently, in such scenarios researchers should, as a minimum standard, report the variability in CIs or SIs constructed across many different minimal-level zonations (see “Methods” and Additional file 1: Fig. S8). Note that where the minimal unit is unmodifiable this variability cannot be examined, representing a limitation that should be acknowledged (see previous paragraph).

To clarify the statistical method we propose, and the theoretical ideas presented above, here we outline a brief algorithm illustrating the steps involved in a practical implementation of our framework:

- *Step 1* Determine the minimal meaningful geographic unit of analysis, by considering:
  - Any limitations of the data gathering method, and
  - Meaningfulness to the process being examined.
- *Step 2* Estimate an association of interest at the minimal level, either:
  - Directly at the minimal level, resulting in a CI for the association of interest, or
  - Indirectly, by constructing a minimal-level SI for the association of interest. This process involves:
    1. Estimating the association at numerous different higher-scale zonations;
    2. Fitting a regression curve to this set of estimates;
    3. Extrapolating the fitted curve to the minimal level, to obtain an EI; and
    4. Assuming the structure of a data-generating process at the minimal level, repeatedly simulating minimal-level data and iterating steps (1)–(3) for each dataset, to construct the minimal-level SI.
- *Step 3* Consider the nature of the minimal-level areal unit:
  - If unmodifiable, and no other meaningful minimal-level zonation could possibly be obtained, then report the CI or SI obtained in step 2.
  - If unmodifiable, and other meaningful minimal-level zonations could theoretically, but cannot currently, be obtained, then:
    1. Report the CI or SI obtained in step 2; and
    2. Acknowledge the limitation introduced by consideration of that single, unmodifiable minimal-level zonation.
  - If modifiable, and other minimal-level zonations can be constructed, then repeat step 2 numerous times using different zonations, and report the different CIs or SIs obtained based on these zonations.

It is well known that smaller bias but larger variance is generally observed when examining smaller units. However, the crux of our argument is that there is a minimal geographical unit at which a true data-generating process is operating, and at which associations should therefore be properly estimated (either by obtaining CIs by directly fitting models at that level or using our SI approach). These ideas are supported by our findings. Therefore, if there is a relatively high level of variability in the data at the minimal level, this will be reflected in the width of the minimal-level CIs or SIs for the effects of interest. While this may be considered to be a drawback of our method, it is necessary and unavoidable in order to properly deal with the MAUP. This is in contrast to the idea that it may be possible to identify an ‘optimal’ areal unit at a scale which is not necessarily the minimal level, possibly by minimising mean squared error (MSE) or by optimising some other metric balancing variance and bias.

Importantly, we note that our example models are intentionally simple in order to convey the proposed method. In particular, we have not considered accounting for spatial autocorrelation. We believe this is reasonable, since it has been previously demonstrated that the MAUP manifests regardless of whether or not spatial autocorrelation exists in a particular dataset [5], and thus also regardless of whether or not it is accounted for in fitted statistical models. However, in practice, more complex models, adjusting for multiple covariates and accounting for spatial autocorrelation, for example, may be appropriate. These additional parameters can be incorporated into the framework we have described. In particular, with regards to spatial autocorrelation, this might be included if some covariates had not been fitted or were not able to be observed. In such cases, spatial autocorrelation can be considered to be a type of surrogate variable, and accounting for it can be used as a means of correcting for the missing variables [33]. However, this would not always be done, for example when accounting for spatial dependence is not relevant to the research question [24], or when there is no evidence that it is present.

Our method can be extended to scenarios where the minimal unit is something other than a geographical area, for example an individual. However, such examples should not be confused with an attempt to extrapolate beyond the minimal level at which data is available (e.g. to individual level when only aggregate data is available) and subsequently to infer conclusions at that extra-minimal level. Researchers have long desired the ability to make such inference, but attempts to do so ignore the loss of information due to aggregation and typically rely on unrealistic assumptions [34]. This principle is described by the ecological fallacy, which is a problem related to, but distinct from, the MAUP [3, 17]. It is worth noting that both the ecological fallacy and the MAUP are special cases of the more general class of ‘change of support problems’ (COSPs) described in the geostatistical literature [23], and that there is potential to extend our framework to other COSPs both within and outside the spatial domain, such as the modifiable temporal unit problem (MTUP) [35].

There is an ongoing debate in the literature regarding whether a solution to the MAUP should be geographical in nature rather than purely mathematical [18]. In the latter case, it has been suggested that methods whose results are dependent on the areal units used should be rejected a priori [36, 37]. Others have argued that addressing the MAUP using methods that disregard the areal units used is nonsensical [1, 38]. This tension is exemplified in the formulations of models including a parameter ‘G’ (i.e. ‘geography’) in [19]. However, in that paper it was acknowledged that estimating ‘G’ would be difficult in practice, and no guidance for doing so was provided. We argue that the consideration of areal units must form part of a solution to the MAUP, since the MAUP only manifests in their presence. Our framework incorporates the geographical characteristics of areal units by combining association estimates from numerous zonations, at multiple geographical scales. This approach embodies previous suggestions that the MAUP should be solved through exploiting its impact [1, 17].

Our results provide a generalized theoretical framework within which previous findings and misconceptions related to the MAUP can be understood. It has been suggested that the MAUP can be avoided by analyzing individual-level data [1], or, more generally, by measuring information at the level of some ‘basic entity’ which is not necessarily spatial [7, 39]. However, this view is not generalizable when a basic entity is not easily defined, or where variables (e.g. density) require spatial specification [32]. Where a basic entity can be defined, this represents a special case of an unmodifiable minimal-level unit of analysis as defined within our framework. One study stated that “in general, estimators from a higher scale analysis will be biased for the equivalent individual-level, or lower scale estimates” [9], but did not provide a statistically robust method to estimate associations at finer scales. Furthermore, we have demonstrated that this is a universal, rather than a general, truth. The idea that variables may be more accurately measured at finer scales has also been recognized where a minimal unit of analysis is modifiable (e.g. in remote sensing; for example see [40]). However, this idea fails to consider the potential importance of using different zonations at the finest scale, which we have investigated. Another study did recognize the importance of considering multiple minimal-level zonations when the minimal unit is modifiable [32], but proposed averaging results based on these multiple zonations. In contrast, previously in this Discussion we have argued that there is no justification for doing so, since no single true effect is shared between differing minimal-level zonations, and minimal-level SIs or CIs will only have asymptotic coverage of the true effect specific to the chosen minimal-level zonation. The existing minimum standard of investigating the sensitivity of a result to the MAUP, by examining multiple zonations at multiple scales [4, 5], is flawed by failing to acknowledge that only estimates at minimal-level zonations are unbiased. It has also been suggested that there may be a particular scale or zonation at which a phenomenon operates and should be appropriately observed [10, 15, 31]. This scenario is described by the minimal meaningful unit of analysis within our framework. Finally, the suggestion that an optimal zonation at some scale can be identified, based on criteria such as maximizing correlation coefficients [1], is incorrect, since any estimate thus obtained will be biased if it is not constructed at the minimal level. Furthermore, in a multivariate context it is impossible to construct a zonation that is optimal for all variables [7].

It could be suggested that extensive investigation of the MAUP and its impact is a purely academic concern. However, the biases introduced by the MAUP, as described in this paper, continue to undermine conclusions based on analyses of spatially aggregated data in the real world, impacting on interventions within fields as diverse as climate change, ecology, health, education, and social inequality. This reality is only properly recognized in a tiny minority of studies (for example see [41, 42]).

## Conclusions

In conclusion, the findings of studies which fail to estimate associations at a minimal geographic unit of analysis are untrustworthy due to the impact of the MAUP. The findings of these studies need to be reconsidered based on our work, since none follow the guidelines for rigorous estimation of associations we have outlined. Continued neglect of the MAUP in studies using spatially aggregated data, given its potential impact, would be scientifically indefensible. However, more positively, future studies estimating associations using spatially aggregated data will now be able to adequately address the MAUP in their analyses using our guidelines, and should do so as a new minimum standard for the analysis of areal data.

## Methods

### Data

#### Perth data (Figs. 1a, b, 3a, 4a)

Population and socioeconomic data for SA1s across the Perth metropolitan region in Western Australia were obtained from the 2011 Australian Census [20]. SA1s are the smallest unit for the release of Census data, having a mean population size of approximately 430 individuals. The socioeconomic measure used was the Index of Relative Socio-economic Advantage and Disadvantage (IRSAD), which summarizes information about the economic and social conditions of people and households within a geographic area, including measures of relative advantage and disadvantage [26]. Areas with high scores on this index have a relatively high incidence of advantage and a relatively low incidence of disadvantage.

MH ED presentations were extracted from the Emergency Department Data Collection (EDDC) [43]. These presentations were identified as having an International Statistical Classification of Diseases and Related Health Problems, Tenth Revision, Australian Modification (ICD-10-AM) code [44] beginning with ‘F’ or a major diagnostic category (MDC) of 19 (‘Mental diseases and disorders’) or 20 (‘Alcohol/drug use and alcohol/drug induced organic mental disorders’).

#### Perth data (Figs. 3d, 4d)

This cohort comprises individuals hospitalized in metropolitan Perth between June 1st 2015 and July 30th 2016, who were aged 55 or above at their first hospitalization in that time period (their ‘index’ hospitalization). Individuals with an ICD-10-AM-coded principal diagnosis of Z49.1 (‘Same-day and overnight episodes of care for dialysis’) at their index hospitalization were excluded.

The Charlson Comorbidity Index (CCI) is a measure of comorbidity originally intended to predict death within twelve months in an acute hospital setting [45], but validated for use within many different contexts. The CCI was adopted for use in conjunction with ICD-9 coding [46] and later for use in conjunction with ICD-10-AM coding [28]. The CCI for each individual was calculated based on comorbidities listed among their additional diagnoses at their index hospitalization combined with the principal diagnoses of any antecedent hospitalizations within 365 days of their index hospitalization. Associated weights for the CCI were calculated based on the method described by Sundararajan et al. [28].

#### London data (Figs. 1c, d, 3b, 4b)

Population and digital boundaries for LSOAs across London were obtained from the 2011 Census in England and Wales [21]. LSOAs are small areas with a mean population size of approximately 1700 residents, or 650 households. There are 32,844 LSOAs in England and 4835 LSOAs in London [47]. The socioeconomic variable used is derived from the 2015 Index of Multiple Deprivation (IMD) for LSOAs across London [27]. The 2015 IMD measures seven dimensions of deprivation: income, employment, health, education, barriers to housing and services, crime, and the quality of the living environment [27]. The individual police-recorded crimes are publicly available via the *police.uk* website, delineated by police force and 2011 LSOA [21]. The crime dimension of the IMD combines police-recorded crime statistics (of all types) for 2016, including burglary, anti-social behavior, bicycle theft, criminal damage and arson, drugs, possession of weapons, public disorder and weapons, public order, robbery, shoplifting, theft from the person, vehicle crime, violence and sexual offences, and other crimes.

#### Georgia data (Figs. 1e, f, 3c, 4c)

Population and per-capita income of black and non-black individuals were obtained from the 2008–2012 American Community Survey 5-year estimates [22]. Data was obtained at the level of CBGs. CBGs are clusters of census blocks and generally have a population size between 600 and 3000 individuals. A CBG usually covers a contiguous area. Census tracts (CTs) comprise at least one CBG and CBGs are uniquely numbered within CTs.

### Automated zonation construction at different scales

The software AZTool [48, 49] was used to construct 100 different zonations at multiple scales in all geographies examined (Perth, London, and Georgia). AZTool is one of a number of automated zone design packages available to researchers [50]. An introduction to and brief history of AZTool, together with a list of references, free download options, and important contacts, can be found at the following location: www.geodata.soton.ac.uk/software/AZTool.

The scales used in each geography (partially listed in sequence form “min:max (step)”) were:

1. For Perth (Fig. 3a, d): 500:5500 (500); 6500; 8000; and 10,000.
2. For London (Fig. 3b): 2000:10,000 (500); 11,000; 12,000; 15,000; and 20,000.
3. For Georgia (Fig. 3c): 2000:10,000 (500); 11,000; 12,000; 15,000; 20,000; 30,000; and 50,000.

AZTool iteratively aggregates minimal ‘building block’ areal units into larger, contiguous polygons (zones) according to user-defined constraints. Resulting configurations of zones are termed ‘zonations’. Different zonations can be constructed using random seeds characterizing starting points for the initial aggregation. The simplest constraint relates to the target population size for each polygon and associated minimum and maximum population thresholds. Previous research used a minimum threshold of 90% of the target population and did not define a maximum threshold [8]. We followed this example but linked the minimum population threshold to the target population via the function:

1 $$ threshold_{{\rm min} } \, = \,a\, \times \,target\;Pop\, + \,b\, \times \,target\;Pop^{2} $$

where $a$ and $b$ are constants obtained by solving the system of equations generated by taking the minimum threshold at the smallest and largest scales to be 60% and 80% of the target population at those scales, respectively:

2a $$ threshold_{\hbox{min} } \, = \,a\, \times \,target\;Pop_{\hbox{min} } \, + \,b\, \times \,target\;Pop_{\hbox{min} }^{2} $$

2b $$ threshold_{\hbox{max} } \, = \,a\, \times \,target\;Pop_{\hbox{max} } \, + \,b\, \times \,target\;Pop_{\hbox{max} }^{2} $$

To illustrate, Fig. 3a shows data for zonations at scales between 500 and 10,000. The minimum threshold end points are calculated as $60\% \; \times \;500\; = \;300$ and $80\% \; \times \;10,000\; = \;8000$. Substituting these values into Eq. (2) yields:

3a $$ 300\, = \,a\, \times \,500\, + \,b\, \times \,500^{2} $$

3b $$ 8000\, = \,a\, \times \,10{,}000\, + \,b\, \times \,10{,}000^{2} $$

Solving Eq. (3) for $a$ and $b$ results in the following equation, which is then used to calculate the minimum threshold at any target population between 500 and 10,000:

4 $$ threshold_{{\rm min} } \, = \,0.589\, \times \,target\;Pop\, + \,0.0000211\, \times \,target\;Pop^{2} $$

The final zonation output by AZTool for a given random seed is controlled by the number of runs of the initial random aggregation and optimization phases, which we set equal to 50, and the number of swapping cycles to make in each run, which we set to 10. Following [8], we did not utilize any other constraints in generating zonations.

### Estimating the probability of repeat appearance in a high-rate/low-income area

This section describes the calculation of the probabilities presented in conjunction with Fig. 1, using data in Fig. 1a, b as an exemplar. For SA1s appearing in a high-rate area in zonation $z_{i}$, the probability of appearing in a high-rate area in a new, different zonation of the same data (zonation $z_{j}$) may be estimated as:

5 $$ p_{j|i} = \frac{{total\;number\;of\;high\; - \;rate\;SA1s\;common\;to\;zonations\;z_{i} \;and\;z_{j} }}{{total\;number\;of\;high\; - \;rate\;SA1s\;in\;zonation\;z_{i} }} $$

Then, for a particular SA1 appearing in a high-rate area in a particular zonation $z_{i}$, the average probability $p_{.|i}$ of that SA1 appearing in a high-rate area in any other, different zonation may be calculated as the mean of $p_{j|i}$ over all $j \ne i$. Additionally, the uncertainty in the resulting probabilities may be represented by the 2.5% and 97.5% quantiles from the distribution of all values $p_{.|i}$.

Note that while a selected administrative zonation is displayed in Fig. 1a, c (SA2s and MSOAs, respectively), no equivalent zonation was available for Georgia (the obvious choice in terms of population size was CTs, but these do not always comprise contiguous CBGs). Therefore the first zonation out of the 100 zonations underlying Fig. 1f was selected to be displayed in Fig. 1e. The areas in this zonation are referred to simply as ‘areas’ in the legend of Fig. 1 and when describing Fig. 1e, f in the main text.

### Construction of 95% simulation intervals (SIs)

To illustrate how a 95% SI is constructed, a single dataset at SA1 level was simulated based on a NB model fitted to SA1-level data underlying Fig. 3a. This model had parameter estimates of − 3.97 for the intercept ($\alpha$), − 0.0144 (RR = 0.9857) for the single covariate $\beta$ (IRSAD percentile), and 2.7851 for dispersion ($\theta$). These values were used to generate the simulated dataset; thus, the estimate for $\beta$ of − 0.0144 represents the known true value of the effect of interest, $\beta$, underlying the simulated dataset.

The simulated data was aggregated by combinations of scale and zonation, NB models were fitted at each combination, and the set of estimates for $\beta$ from these models was plotted (Additional file 1: Fig. S1). The true effect is indicated, along with the estimated coefficient and 95% CI from a NB model fitted at SA1 level (blue). Note that this estimate is different from the SA1-level estimate fitted to the original dataset underlying Fig. 3a, which was subsequently used to generate the simulated dataset as described. Finally, a 95% SI at SA1 level is shown (red), which is constructed as follows:

1. Regression lines are fitted to the set of parameter estimates from the NB models, i.e. for parameters $\beta$, $\alpha$, $\theta$ respectively (Additional file 1: Fig. S2a–c). Quadratic curves were considered reasonable and were fitted for all three parameters. For each parameter both axes are log-transformed to prevent negative values when extrapolating fitted regression lines to SA1 level.
2. The fitted lines are used to obtain extrapolated intercepts at SA1 level for the three parameters (denoted $EI_{\beta } ,\;EI_{\alpha }$, and $EI_{\theta }$). These had values − 0.0139 (RR = 0.986), − 3.981 and 0.9682 ($\theta$ = 2.6332), respectively.
3. For values $V$ nearby $EI_{\beta }$, data is repeatedly simulated from a NB distribution using the values $EI_{\alpha }$ and $EI_{\theta }$. Each dataset $i \left( {i\; = \;1 , \ldots , 500} \right)$ is aggregated by combinations of scale and zonation, and a NB model is fitted at each combination to form a set of estimates. A quadratic curve is fitted to each set of estimates and extrapolated to SA1 level to form a density of extrapolated intercepts $EI_{\beta i|V}$ corresponding to the value $V$. This process is repeated until two values $A$ and $B$ are found whose quantile values $EI_{\beta ,97.5|A}$ and $EI_{\beta ,2.5|B}$ simultaneously equal $EI_{\beta }$. In this manner convergence to a 95% SI for the original estimate $\beta$ at SA1 level is achieved. The values $A$ and $B$ are the lower and upper bounds of the SI, respectively.

Additional file 1: Fig. S2d shows the densities of estimates $EI_{\beta ,97.5|A}$ and $EI_{\beta ,2.5|B}$. A convergence threshold equal to $0.1\% \times |EI_{\beta } |$ was used to balance computational efficiency of the simulation process and the precision of the obtained 95% SI. Data in Additional file 1: Fig. S2a is re-presented in Additional file 1: Fig. S2e, now including the 95% SI, and again in Additional file 1: Fig. S2f after reversing the log-transformation on both axes (i.e. on the RR scale). Note that Additional file 1: Fig. S2f is similar to Additional file 1: Fig. S1; this was intentional to facilitate the explanation above.

### Probabilistic proof for asymptotic coverage of 95% SIs

By construction a 95% SI obtained using our method has 95% coverage of the minimal-level true effect underlying a particular dataset. Probabilistic reasoning for this assertion is given here. Assuming the selection of an appropriate model structure underlying the data at the minimal level, and assuming that values $EI_{\beta |Z}$ may be sampled for an underlying effect $Z$, which may represent the true effect underlying a particular dataset (denoted $Z_{true}$), or some other value, then:

6 $$ \begin{aligned} & P\left( {EI_{{\beta |Z_{true} }} < EI_{{\beta ,2.5|Z_{true} }} } \right) = 0.025 \\ & P\left( {EI_{{\beta |Z_{true} }} > EI_{{\beta ,97.5|Z_{true} }} } \right) = 0.025 \\ \end{aligned} $$

Furthermore, by construction of the 95% SIs:

7 $$ EI_{\beta ,97.5|A} = EI_{{\beta |Z_{true} }} = EI_{\beta ,2.5|B} $$

where $A$ and $B$ are the bounds of the 95% SI. Assuming that the quantiles of the densities of extrapolated values nearby $EI_{\beta }$ are monotone increasing in the vicinity of $EI_{\beta }$, then substituting Eq. (7) into Eq. (6) gives:

8 $$ \begin{aligned} & P\left( {EI_{\beta ,97.5|A} > EI_{{\beta ,97.5|Z_{true} }} } \right) = 0.025,\;and \\ & P\left( {EI_{\beta ,2.5|B} < EI_{{\beta ,2.5|Z_{true} }} } \right) = 0.025 \\ \end{aligned} $$

It follows that:

9 $$ \begin{aligned} & P\left( {A > Z_{true} } \right) = 0.025,\;and \\ & P\left( {B < Z_{true} } \right) = 0.025 \\ \end{aligned} $$

That is:

10 $$ P\left( {A < Z_{true} < B} \right) = 0.95 $$

This derivation holds for the one-parameter case. If there are additional nuisance parameters in the model (for example $\alpha$ and $\theta$ in Additional file 1: Figs. S1–S2, or spatial autocorrelation, if this is considered) then the above distributions will depend on them, but the results should hold approximately as long as consistent estimates for these nuisance parameters are used. In situations where consistent estimates of nuisance parameters are not available, an adjustment to the 95% SI for $\beta$ is necessary to account for the additional variability arising due to this uncertainty. This scenario represents a special case of a broader multivariate scenario, where SIs may be required for $p$ parameters of interest ($\beta_{1}$, $\beta_{2}$,…, $\beta_{p}$), possibly including several covariates. Future work will generalize the construction of SIs to the multivariate case. Finally, note that this proof has been presented based on coverage of 95%, but it applies equally to other coverage levels which may be desired (e.g. 90%).

### Comment on why SIs are necessary and traditional uncertainty intervals are inappropriate

Several approaches to constructing uncertainty intervals at the minimal level were investigated, including prediction intervals (PIs) and a block bootstrap approach. The bootstrap is a common resampling approach which can be used in the analysis of clustered data [51], for example grouped areal unit data. Our implementation of the block bootstrap approach involved resampling minimal areal units within larger zones before re-estimating association effects at combinations of scale and zonation. However, inconsistent results were observed for all alternative approaches examined, both in terms of bias and in terms of coverage of underlying true effects in simulation studies. Furthermore, the results from these methods were highly dependent on the chosen stopping point and the choice of regression line for different datasets. As noted previously, these inconsistent results can be explained by the fact that $EI_{\beta }$ is merely an interim value used in the construction of a SI for an effect $\beta$. In fact, perhaps counterintuitively, in extreme cases the SI for a particular set of estimates may not necessarily include the value $EI_{\beta }$. Thus, since $EI_{\beta }$ is not an estimate of $\beta$, it is unsurprising that methods involving the construction of uncertainty intervals around $EI_{\beta }$ are not useful.

### Testing our method in an extreme scenario

To demonstrate the strength of our approach, Additional file 1: Fig. S3 describes the construction of a 95% SI for the simulated dataset underlying Additional file 1: Figs. S1–S2, after applying an extreme hypothetical stopping point population size of 8000. After log-transforming both axes, straight lines were necessarily fitted to the set of estimates for parameters $\alpha$, $\beta$, and $\theta$ since estimates at only two scales were available (Additional file 1: Fig. S3a–c). Additional file 1: Fig. S3d shows the densities of $EI_{\beta |A}$ and $EI_{\beta |B}$ following the convergence to a 95% SI for $\beta$. Remarkably, a sensible 95% SI is still obtained, despite data being available at only two scales which were distant from the minimal level. The data in Additional file 1: Fig. S3a is re-presented in Additional file 1: Fig. S3e with the 95% SI shown, and again in Additional file 1: Fig. S3f on the RR scale.

### Description of the construction of 95% SIs for examples shown in Fig. 4

#### Perth data (Fig. 4a)

Additional file 1: Fig. S4, describes the construction of the 95% SI shown in Fig. 4a. Additional file 1: Fig. S4a–c show the set of estimates for the effect ($\beta$), the intercept ($\alpha$), and the dispersion parameter ($\theta$), from NB models fitted at each combination of scale and zonation. After log-transforming both axes, quadratic fits were considered reasonable in each case. Data was repeatedly simulated at SA1 level based on a NB distribution incorporating the extrapolated intercept values $EI_{\alpha }$ and $EI_{\theta }$. Additional file 1: Fig. S4d shows the densities of the values $EI_{\beta |A}$ and $EI_{\beta |B}$ corresponding to the upper and lower bounds (values $A$ and $B$) of the 95% SI constructed for $\beta$ at SA1 level. Additional file 1: Fig. S4e re-presents data in Additional file 1: Fig. S4a with the 95% SI shown, along with the SA1-level estimate and its 95% CI. Additional file 1: Fig. S4f re-presents data in Additional file 1: Fig. S4e on the RR scale. Note that data in Additional file 1: Fig. S4f is equivalent to data in Additional file 1: Fig. S4a.

#### London data (Fig. 4b)

Additional file 1: Fig. S5 describes the construction of the 95% SI shown in Fig. 4b. Additional file 1: Fig. S5a–c show the set of estimates for the effect ($\beta$), the intercept ($\alpha$), and the dispersion parameter ($\theta$), from NB models fitted at each combination of scale and zonation. After log-transforming both axes, quadratic fits were considered reasonable in each case. Data was repeatedly simulated at LSOA level based on a NB distribution incorporating the extrapolated intercept values $EI_{\alpha }$ and $EI_{\theta }$. Additional file 1: Fig. S5d shows the densities of values $EI_{\beta |A}$ and $EI_{\beta |B}$ corresponding to the upper and lower bounds (values $A$ and $B$) of the 95% SI constructed for $\beta$ at LSOA level. Additional file 1: Fig. S5e re-presents data in Additional file 1: Fig. S5a with the 95% SI shown, along with the SA1-level estimate and its 95% CI. Additional file 1: Fig. S5f re-presents data in Additional file 1: Fig. S5e on the RR scale. Note that data in Additional file 1: Fig. S5f is equivalent to data in Fig. 4b.

#### Georgia data (Fig. 4c)

Additional file 1: Fig. S6 describes the construction of the 95% SI in Fig. 4c. Additional file 1: Fig. S6a–c show the set of estimates for the effect ($\beta$), the intercept ($\alpha$), and the residual standard error (RSE), from standard linear models fitted at each combination of scale and zonation. These models incorporated weights to account for differences in population size between polygons, resulting in inflated RSE estimates (Additional file 1: Fig. S6c) which were later divided by the square root of the minimal-level (CBG) population vector when simulating data at that level. After log-transforming the x-axis in all cases and the y-axis for the RSE, quadratic fits were considered reasonable in each case. Data was repeatedly simulated at CBG level based on a standard linear model incorporating the extrapolated intercept values $EI_{\alpha }$ and $EI_{RSE}$. Additional file 1: Fig. S6d shows the densities of values $EI_{\beta |A}$ and $EI_{\beta |B}$ corresponding to the upper and lower bounds (values $A$ and $B$) of the 95% SI constructed for $\beta$ at CBG level. Additional file 1: Fig. S6e re-presents data in Additional file 1: Fig. S6a with the 95% SI shown, along with the CBG-level estimate and its 95% CI. Note that data in Additional file 1: Fig. S6e is equivalent to data in Fig. 4c.

#### Perth data (Fig. 4d)

Additional file 1: Fig. S7 describes the construction of the 95% SI shown in Fig. 4d. Additional file 1: Fig. S7a–b show the set of estimates for the effect ($\beta$) and the intercept ($\alpha$), from standard linear models fitted at each combination of scale and zonation. Note that new zonations were not constructed for the hospitalized cohort, rather, the mean polygon population size (hospitalized cohort) on the x-axis represents the mean number of hospitalized individuals residing in the zonations constructed using the total census population (Additional file 1: Fig. S1). Quadratic fits were considered reasonable in each case. Data was repeatedly simulated at individual level based on a Poisson distribution incorporating the extrapolated intercept value $EI_{\alpha }$. A Poisson model was necessary because the Charlson comorbidity index is discretized at individual level. Additional file 1: Fig. S7c shows the densities of values $EI_{\beta |A}$ and $EI_{\beta |B}$ corresponding to the upper and lower bounds (values $A$ and $B$) of the 95% SI constructed for $\beta$ at individual level. Additional file 1: Fig. S7d re-presents data in Additional file 1: Fig. S7a with the 95% SI shown, along with the individual-level estimate and its 95% CI. Note that data in Additional file 1: Fig. S7d is equivalent to data in Fig. 4d.

### Method to present results for a modifiable minimal unit of analysis

If it is assumed that data in Fig. 2a was measurable at a finer scale than that presented, but was not considered meaningful at that scale, then different configurations of the minimal level zonation could be constructed. For example, suppose each minimal unit comprised 50 × 50 smaller cells. Therefore, the minimal-level grid could be shifted 49 times in one direction without repeating a previous configuration. Considering only single-direction shifts, i.e. to the right or upwards, a total of 99 minimal-level model estimates of the association between variables $A$ and $B$ can be obtained [including the estimate corresponding to the original minimal-level configuration (Fig. 2a)]. Additional file 1: Fig. S8 shows these 99 estimates along with their 95% CIs, ordered by the value of the point estimates. The null effect of zero is indicated by a horizontal grey line and the effect of − 0.86 corresponding to the configuration in Fig. 2a is indicated by a heavy vertical line.

Clearly, a wide range of estimates are obtainable when the minimal unit of analysis is modifiable. In fact, as this example shows, the estimate for a particular variable may range from statistically negative to statistically positive. Therefore, it is imperative that researchers construct similar plots when conducting analyses based on a modifiable minimal unit, in order to demonstrate the reproducibility of estimates across different minimal-level zonations. Here, there is no reproducibility in either the direction of the association or in the magnitude of the estimates; results showing a consistent direction and magnitude of association would be far more reliable and defensible. Finally, we note that in cases where minimal-level models cannot be fitted, SIs should be constructed and presented in place of CIs when following this approach.

## Additional file

**Additional file 1.** Figures S1–S8.

## Abbreviations

CBG: census block group
CCI: Charlson comorbidity index
CI: confidence interval
COSP: change of support problem
CT: census tract
EDDC: emergency department data collection
EI: extrapolated intercept
ICD-10-AM: international statistical classification of diseases and related health problems, tenth revision, Australian modification
IMD: index of multiple deprivation
IRSAD: index of relative socio-economic advantage and disadvantage
LSOA: lower layer super output area
MAUP: modifiable areal unit problem
MDC: major diagnostic category
MH ED: mental health-related emergency department
MSOA: middle layer super output area
MSE: mean squared error
MTUP: modifiable temporal unit problem
NB: negative binomial
SA1: statistical area level 1
SA2: statistical area level 2
RR: rate ratio
RSE: residual standard error
SI: simulation interval
