# Supplementary material for: Incorporating geography into a new generalized theoretical and statistical framework addressing the modifiable areal unit problem
Source: Int J Health Geogr. 2019 Mar 27;18:6. doi: 10.1186/s12942-019-0170-3 (PMC6437958; doi:10.1186/s12942-019-0170-3)

**Additional file 1.**

**Fig. S1. A minimal-level 95% SI describes the impact of the MAUP for a particular geography.** Estimated RRs of MH ED presentations associated with a unit increase in IRSAD percentile, from models fitted to a simulated dataset aggregated to different combinations of scale and zonation. The known true effect is indicated, along with the SA1-level estimate and its 95% CI (blue), a fitted line, the extrapolated intercept for the effect$\beta$ (${EI}_{\beta}$), and the 95% SI for $\beta$ (red).


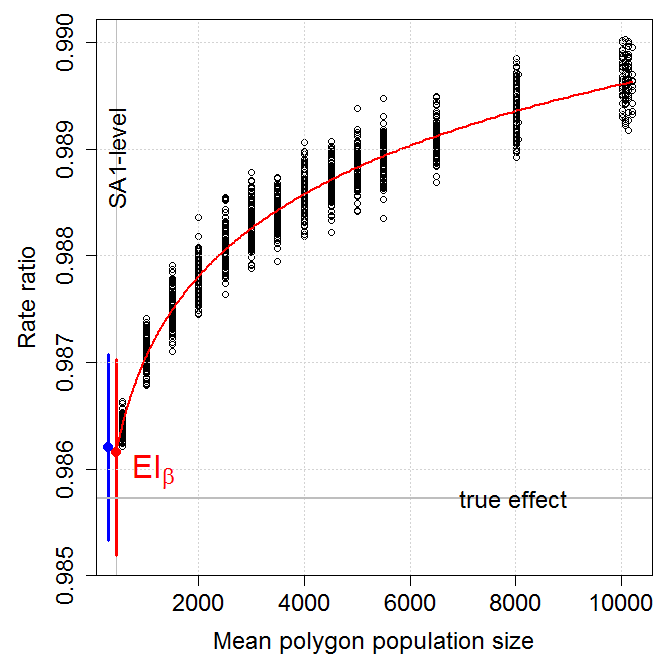


**Fig. S2. 95% SI for a simulated dataset with known true effect and no stopping-point population size. (a** to **c)** Fitted curves for parameters $\beta$, $\alpha$, and $\theta$, respectively, with corresponding extrapolated intercepts ${EI}_{\beta}$, ${EI}_{\alpha}$, and ${EI}_{\theta}$ at SA1 level. **(d)** Densities of values ${EI}_{\beta|A}$ and ${EI}_{\beta|B}$ corresponding to the bounds of the 95% SI constructed for $\beta$ at SA1 level. **(e)** Data in (a) re-presented with the 95% SI shown (red) along with the SA1-level estimate and its 95% CI (blue). **(f)** Data in (e) re-presented on the RR scale.

**
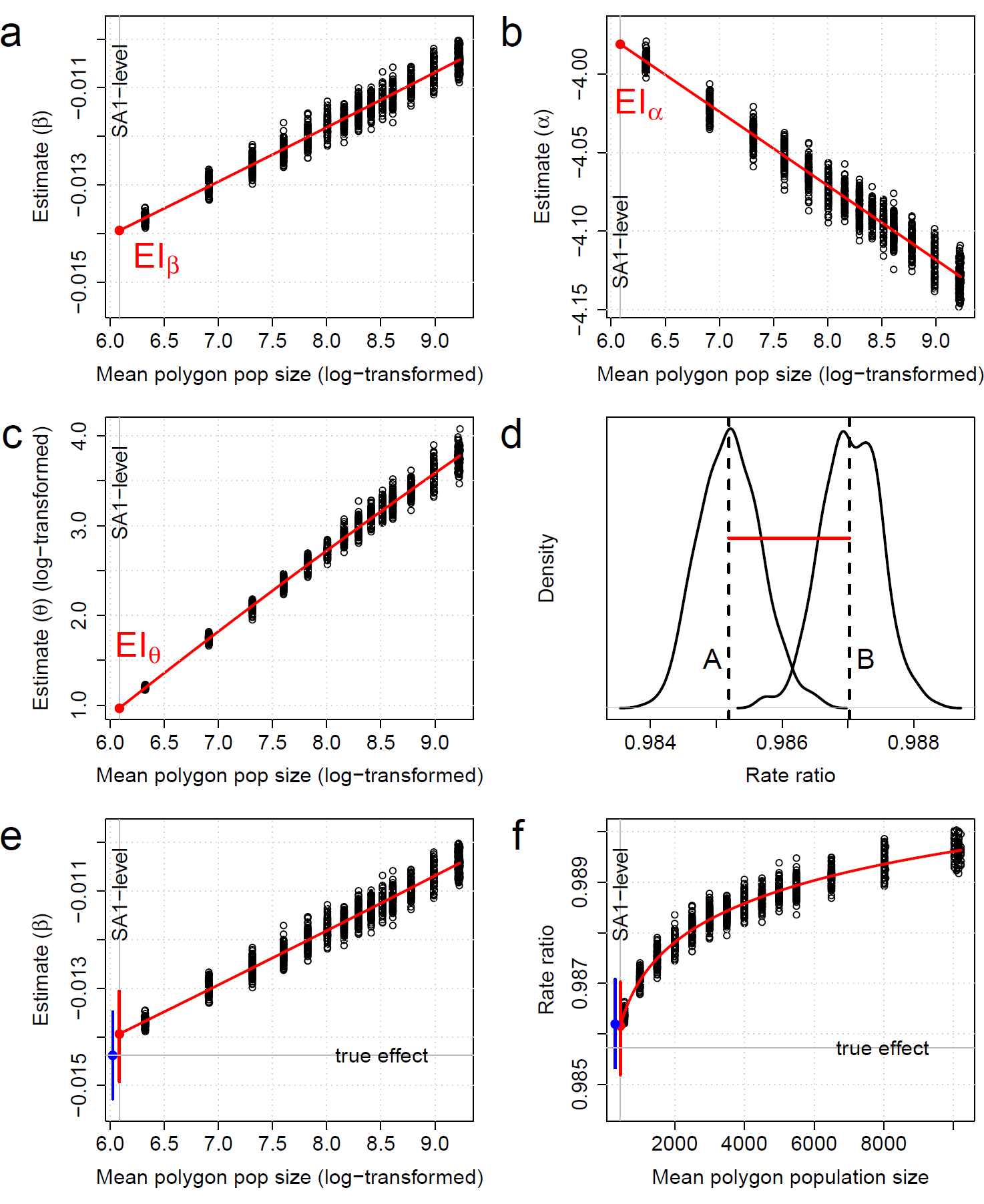
**

**Fig. S3. 95% SI for a simulated dataset with known true effect and stopping-point population size 8,000. (a** to **c)** Fitted curves for parameters $\beta$, $\alpha$, and $\theta$, respectively, with corresponding extrapolated intercepts ${EI}_{\beta}$, ${EI}_{\alpha}$, and ${EI}_{\theta}$ at SA1 level. **(d)** Densities of values ${EI}_{\beta|A}$ and ${EI}_{\beta|B}$ corresponding to the bounds of the 95% SI constructed for $\beta$ at SA1 level. **(e)** Data in (a) re-presented with the 95% SI shown (red) along with the SA1-level estimate and its 95% CI (blue). **(f)** Data in (e) re-presented on the RR scale (y-axis).

**
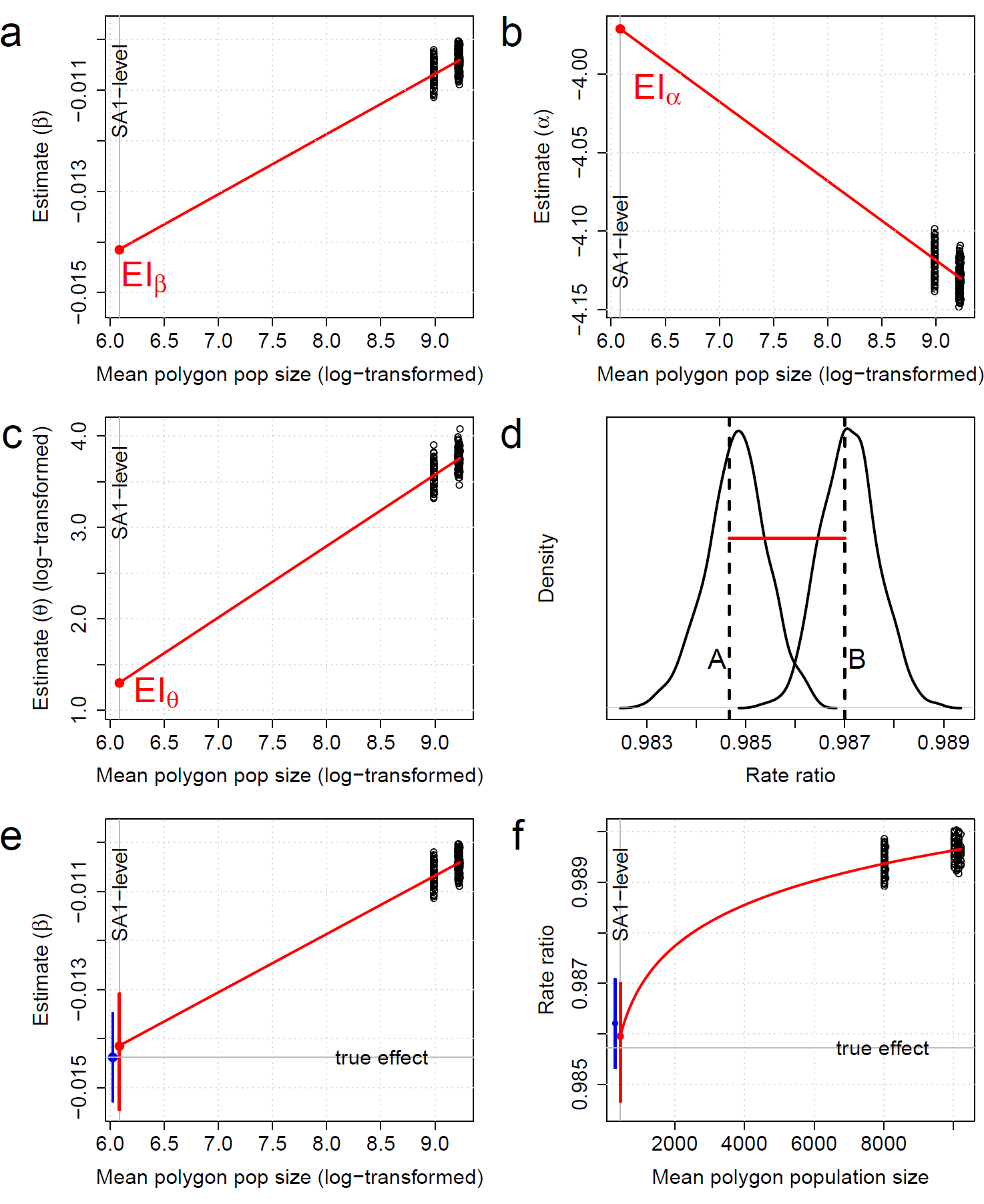
**

**Fig. S4. 95% SI for data in Fig. 3a with stopping-point population size 2,000.** **(a** to **d)** Fitted curves for parameters $\beta$, $\alpha$, and $\theta$, respectively, with corresponding extrapolated intercepts ${EI}_{\beta}$, ${EI}_{\alpha}$, and ${EI}_{\theta}$ at SA1 level. **(d)** Densities of values ${EI}_{\beta|A}$ and ${EI}_{\beta|B}$ corresponding to the bounds of the 95% SI for $\beta$ at SA1 level. **(e)** Data (a) re-presented with the 95% SI shown (red) along with the SA1-level estimate and its 95% CI (blue). **(f)** Data in (e) re-presented on the RR scale.

**
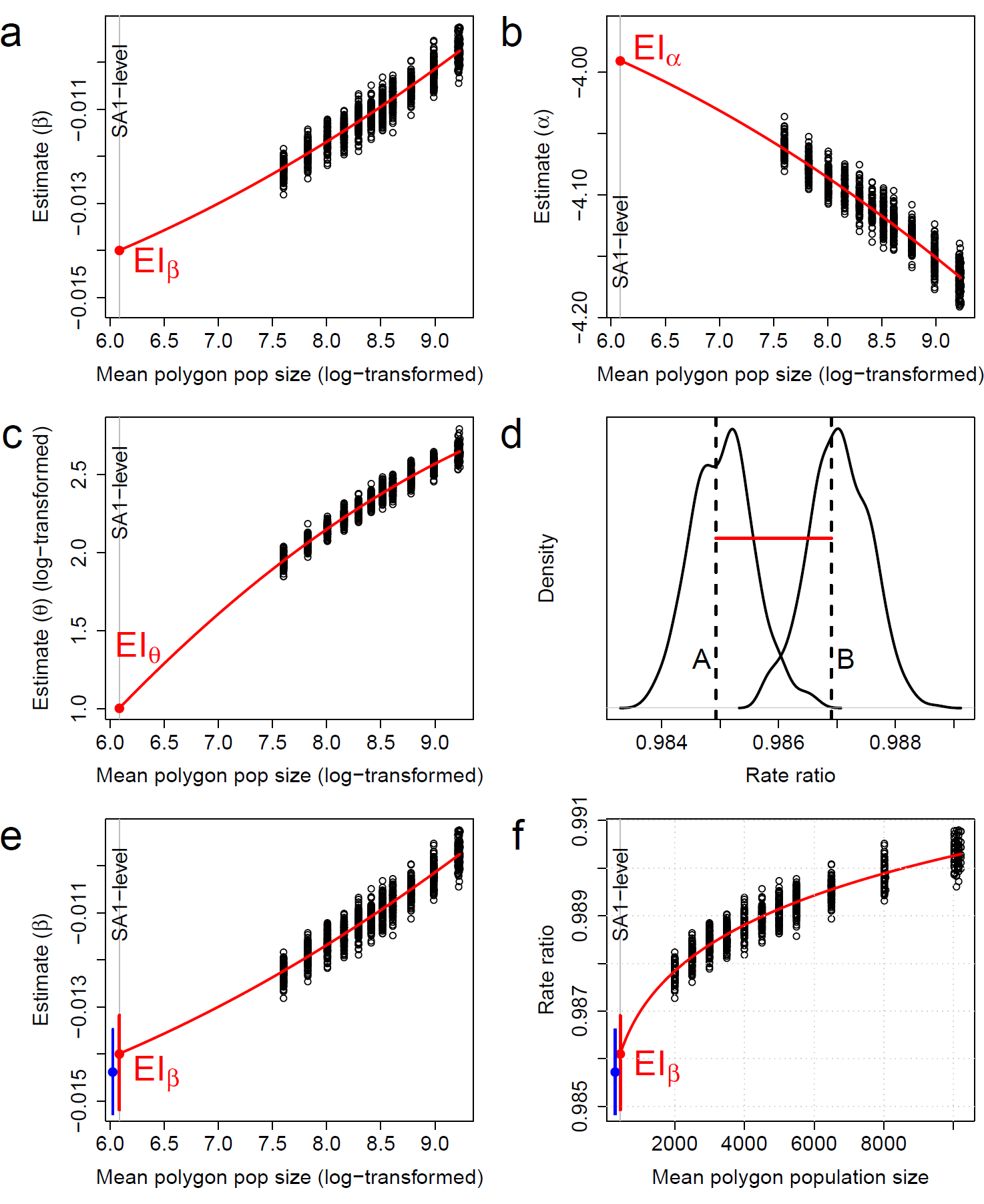
**

**Fig. S5. 95% SI for data in Fig. 3b with stopping-point population size 3,000. (a** to **d)** Fitted curves for parameters $\beta$, $\alpha$, and $\theta$, respectively, with corresponding extrapolated intercepts ${EI}_{\beta}$, ${EI}_{\alpha}$, and ${EI}_{\theta}$ at LSOA level. **(d)** Densities of values ${EI}_{\beta|A}$ and ${EI}_{\beta|B}$ corresponding to the bounds of the 95% SI for $\beta$ at LSOA level. **(e)** Data in (a) re-presented with the 95% SI shown (red) along with the LSOA-level estimate and its 95% CI (blue). **(f)** Data in (e) re-presented on the RR scale.

**
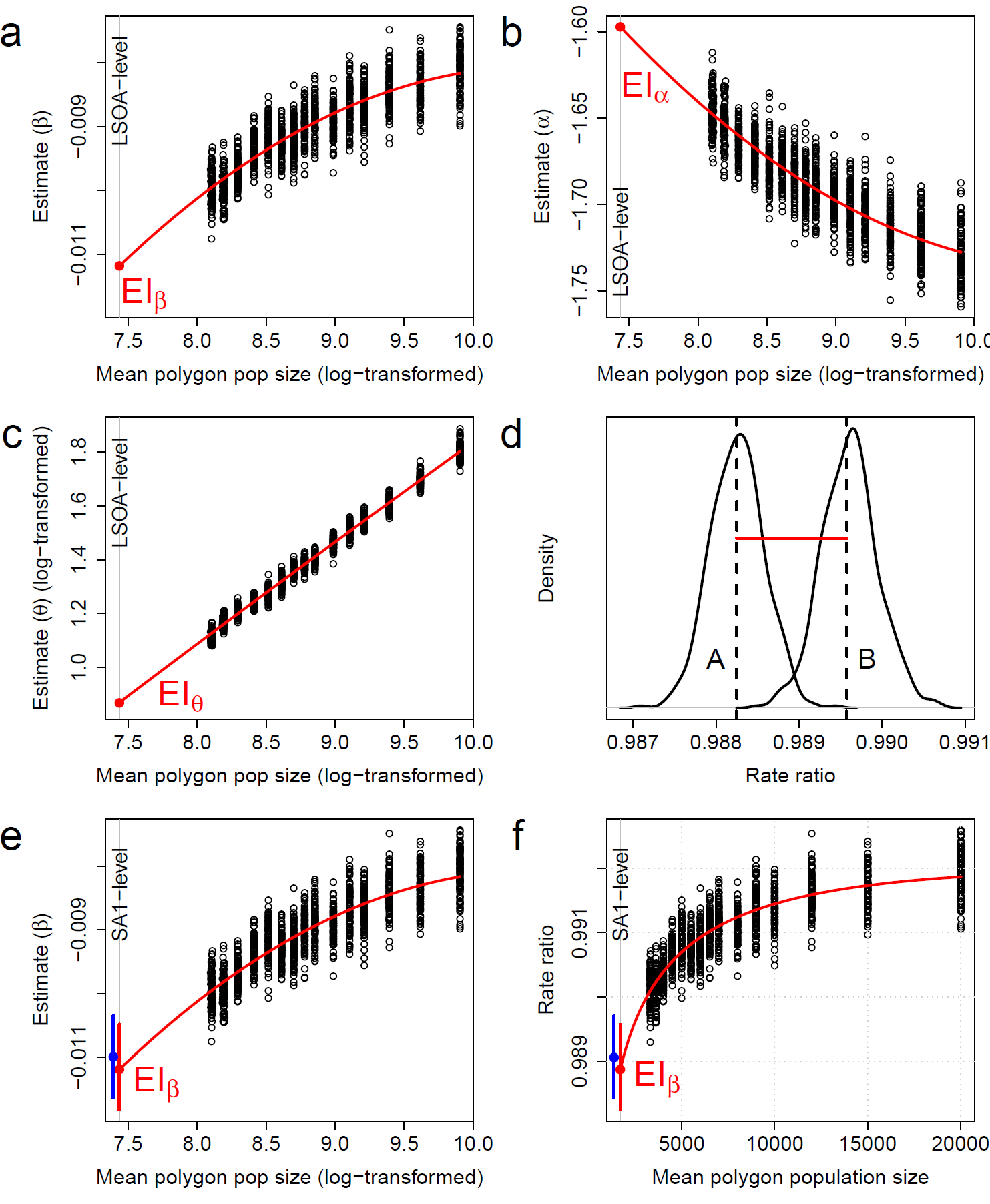
**

**Fig. S6. 95% SI for data in Fig. 3c with stopping-point population size 3,000. (a** to **c)** Fitted curves for parameters $\beta$ and $\alpha$, respectively, and for $RSE$, with corresponding extrapolated intercepts ${EI}_{\beta}$, ${EI}_{\alpha}$, and ${EI}_{RSE}$ at CBG level. **(d)** Densities of values ${EI}_{\beta|A}$ and ${EI}_{\beta|B}$ corresponding to the bounds of the 95% SI for $\beta$ at CBG level. **(e)** Data in (a) re-presented with the 95% SI shown (red) along with the CBG-level estimate and its 95% CI (blue).

**
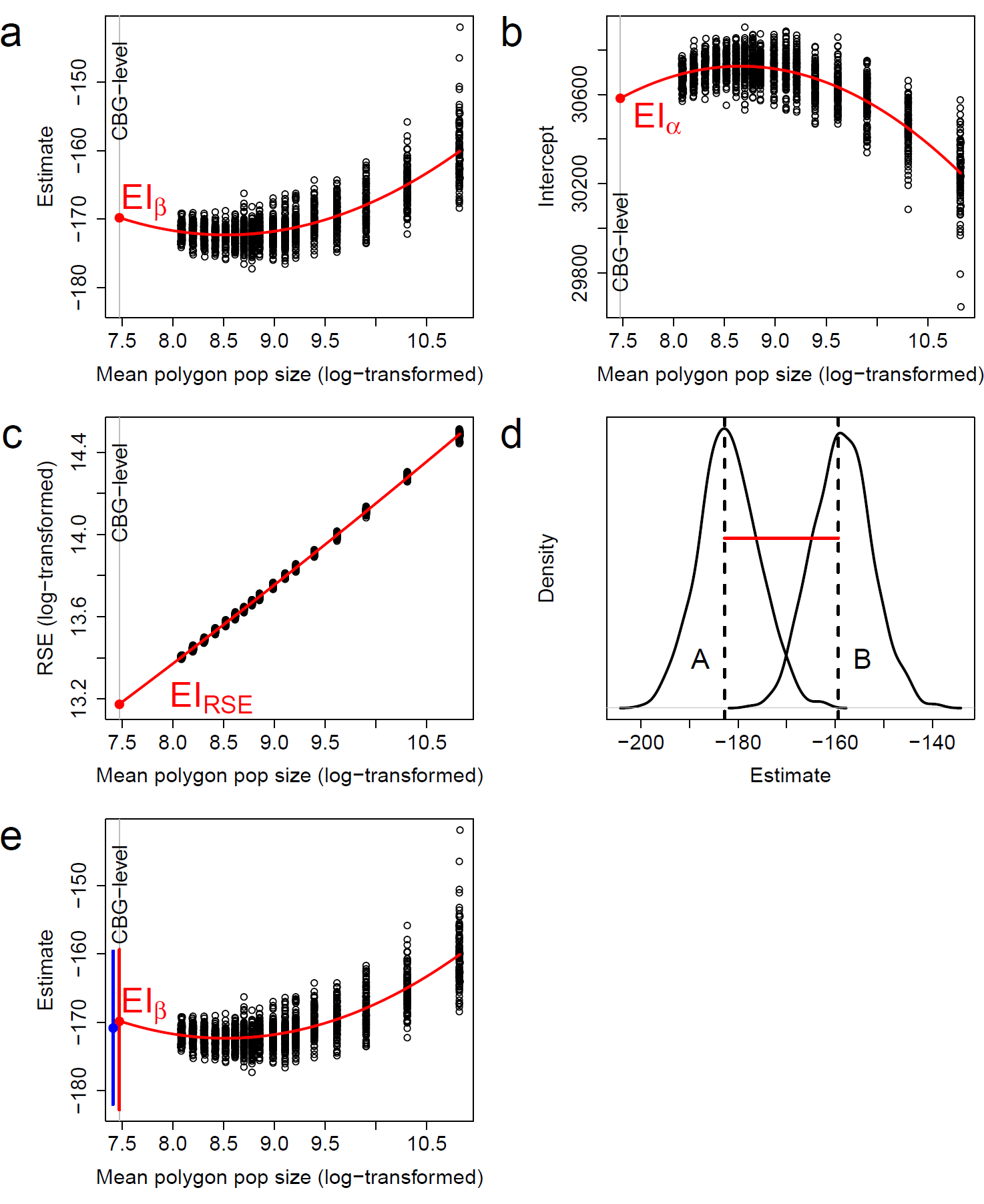
**

**Fig. S7. 95% SI for data in Fig. 3d with stopping-point population size 2,000. (a** to **b)** Fitted curves for parameters $\beta$ and $\alpha$, respectively, with corresponding extrapolated intercepts ${EI}_{\beta}$ and ${EI}_{\alpha}$ at individual level. **(c)** Densities of values ${EI}_{\beta|A}$ and ${EI}_{\beta|B}$ corresponding to the bounds of the 95% SI for $\beta$ at individual level. **(d)** Data in (a) re-presented with the 95% SI shown (red) along with the individual-level estimate and its 95% CI (blue).


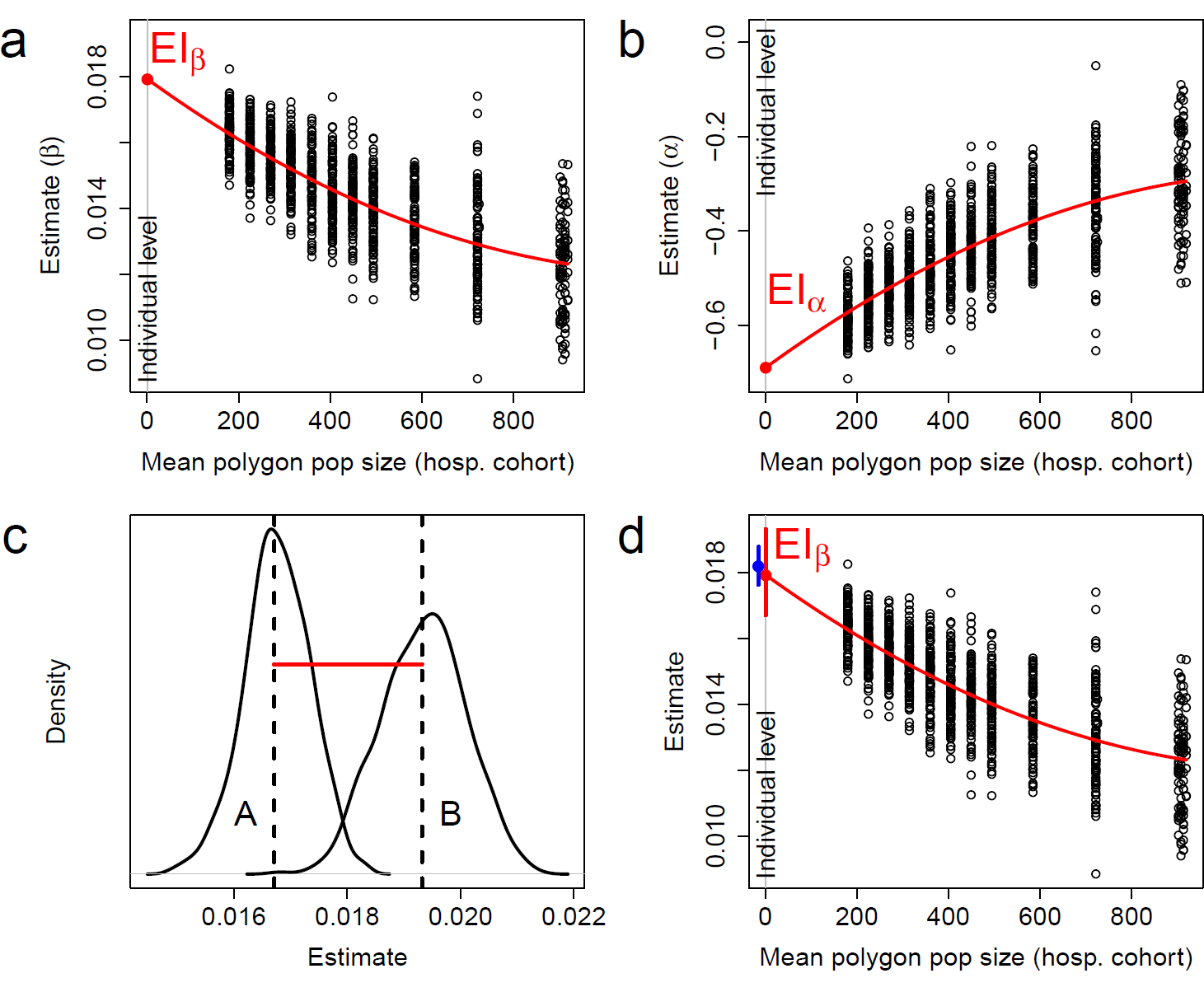


**Fig. S8. Estimates and CIs from models fitted at the minimal level to data underlying Fig. 2.** Point estimates and 95% CIs corresponding to different configurations of a minimal meaningful raster cell size. The null effect and the estimate and CI corresponding to the layout shown in Fig. 2a are indicated by a horizontal line and a heavy vertical line, respectively.


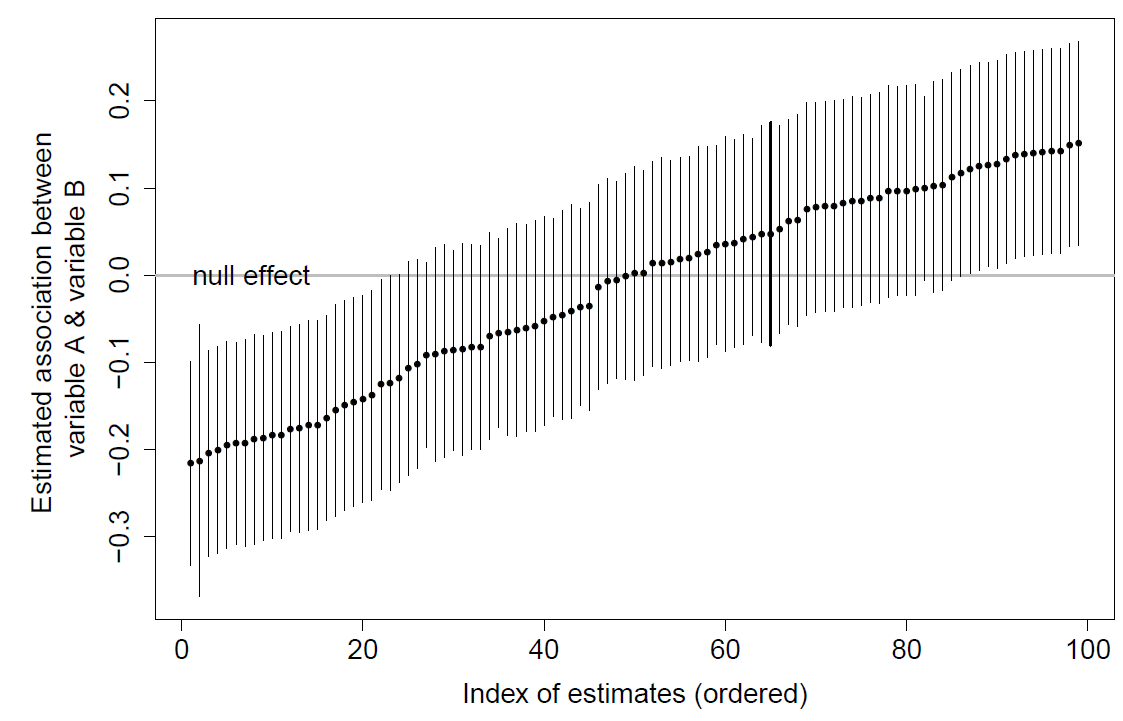

Supplement: Supplementary file 1 — Additional file 1. Figures S1–S8. [file 12942_2019_170_MOESM1_ESM.docx]
